# Tree‐centric mapping of forest carbon density from airborne laser scanning and hyperspectral data

**DOI:** 10.1111/2041-210X.12575

**Published:** 2016-05-14

**Authors:** Michele Dalponte, David A. Coomes

**Affiliations:** ^1^ Department of Sustainable Agro‐ecosystems and BioresourcesResearch and Innovation CentreFondazione E. MachVia E. Mach 138010San Michele all'Adige (TN)Italy; ^2^ Forest Ecology and Conservation GroupDepartment of Plant SciencesUniversity of CambridgeDowning StreetCambridgeCB2 3EAUK

**Keywords:** above‐ground biomass, airborne laser scanning, carbon density, hyperspectral imaging, individual tree crowns, LIDAR, temperate forests

## Abstract

1. Forests are a major component of the global carbon cycle, and accurate estimation of forest carbon stocks and fluxes is important in the context of anthropogenic global change. Airborne laser scanning (ALS) data sets are increasingly recognized as outstanding data sources for high‐fidelity mapping of carbon stocks at regional scales.
2. We develop a tree‐centric approach to carbon mapping, based on identifying individual tree crowns (ITCs) and species from airborne remote sensing data, from which individual tree carbon stocks are calculated. We identify ITCs from the laser scanning point cloud using a region‐growing algorithm and identifying species from airborne hyperspectral data by machine learning. For each detected tree, we predict stem diameter from its height and crown‐width estimate. From that point on, we use well‐established approaches developed for field‐based inventories: above‐ground biomasses of trees are estimated using published allometries and summed within plots to estimate carbon density.
3. We show this approach is highly reliable: tests in the Italian Alps demonstrated a close relationship between field‐ and ALS‐based estimates of carbon stocks (*r* ^2^ = 0·98). Small trees are invisible from the air, and a correction factor is required to accommodate this effect.
4. An advantage of the tree‐centric approach over existing area‐based methods is that it can produce maps at any scale and is fundamentally based on field‐based inventory methods, making it intuitive and transparent. Airborne laser scanning, hyperspectral sensing and computational power are all advancing rapidly, making it increasingly feasible to use ITC approaches for effective mapping of forest carbon density also inside wider carbon mapping programs like REDD++.

## Introduction

Forest ecosystems cover about 30% of our planet, contain 80% of the Earth's biomass and account for 75% of the gross primary productivity of the terrestrial biosphere (IPCC, 2006; Pan *et al*. 2013) as well as harbouring much terrestrial biodiversity (Ozanne *et al*. 2003). They account for 50% of the annual carbon flux between the atmosphere and the Earth's land surface (Beer *et al*. 2010), and sequestering carbon equivalent to about 30% of the fossil fuel emissions (Pan *et al*. 2011). Current knowledge about the contributions of forest to global carbon cycling comes primary from field‐based inventory data. Many developed countries have impressive plot networks which provide unbiased and precise national estimates of forest attributes [e.g. >200 000 plots in the USA (Hulshof, Swenson & Weiser 2015)], but remote sensing data are increasingly used to complement these plot networks, including satellite multispectral data, laser scanning and RADAR (Gonzalez *et al*. 2010; Thurner *et al*. 2014).

The most accurate remote sensing technology for monitoring forest carbon is airborne laser scanning (ALS; Lefsky *et al*. 2002; Asner *et al*. 2012). By firing hundreds of thousands of laser pulses per second at land surfaces, and measuring surface elevation within a few centimetres precision, ALS sensors produce highly detailed 3D point clouds pinpointing locations on leaves, branches and the forest floor. Classically, regression techniques have been used to model above‐ground carbon density measured in plots (CD_PLOT_ in Mg C per hectare) as a function of various summary statistics derived from the ALS point cloud; however, a limitation is that these models are site specific (Næsset 2002; Hudak *et al*. 2006). A recent advance has been a recognition that carbon density (CD_PLOT_) can be accurately modelled using:

(eqn 1) $${\text{CD}}_{\mathrm{PLOT}}=a*{\bar{\text{WD}}}^{b}*{\text{BA}}^{c}*{\overset{¯}{H}}^{d},$$

where $\overset{¯}{H}$ is average canopy height obtained from ALS (e.g. mean canopy height or the canopy top height), $\bar{\text{WD}}$ is average wood density (WD) measured on the ground, BA is basal area of a plot, and *a, b*,* c* and *d* are parameters estimated by regression (Asner *et al*. 2012, 2014). Interestingly, a comparison of models developed for four contrasting tropical forests indicates that *d* is approximately constant among sites, suggesting it is a ‘universal’ model for tropical forests. However, eqn 1 cannot be derived by summing individual tree biomasses unless the tree size distribution is known, and relies on inputs from the ground (i.e. mean basal area and mean WD) (Vincent, Sabatier & Rutishauser 2014).

The objective of this study was to develop and test a tree‐centric approach for mapping forest carbon, using a combination of ALS and hyperspectral data, building on research reviewed by Breidenbach & Astrup (2014). The primary benefit of adopting this approach is that it is fundamentally similar to methods already available for analysing forest plot data (e.g. Coomes *et al*. 2001; Chen, Vaglio Laurin & Valentini 2015). Within forest inventories, the approach is to (i) measure the stem diameters and heights of all trees above a certain size threshold within a plot; (ii) use published allometric equations to estimate tree biomasses from these measurements, which, typically, take the form:

(eqn 2) $${\hat{\text{AGB}}}_{\mathrm{TREE}}=\alpha *{\text{WD}}^{\beta }*{\text{DBH}}^{\gamma }*H^{\delta },$$

where ${\hat{\text{AGB}}}_{\mathrm{TREE}}$ is the estimated above‐ground biomass in kilograms of a tree, *H* its height in m, DBH its diameter at breast height in cm, WD its wood density in g cm^−3^, and α, β, γ, δ are regression coefficients available in published papers (e.g. Chave *et al*. 2014); (iii) sum up the individual biomasses within the plot; and (iv) convert plot‐level biomass estimates to carbon densities by multiplying by carbon content values. Here, we follow a similar approach, except that instead of visiting plots and measuring trees by hand, we (i) use algorithms to detect individual trees from airborne imagery then estimates the height and crown area of each detected tree and then use regression relationships to estimate DBH from these measurements; after that steps (ii–iv) are exactly the same as above. Ground‐based studies have shown that *D* ∝ *f*(*H*, CA), where CA is the crown area and *H* is the height of the tree (Coomes *et al*. 2012; Rüger & Condit 2012). Thus, eqn 2 can be transformed into:

(eqn 3) $${\hat{\text{AGB}}}_{\mathrm{TREE}}=\alpha *{\text{WD}}^{\beta }*{\left[f\left(H,\text{CA}\right)\right]}^{\gamma }*H^{\delta }.$$

It is increasingly common to collect high‐spatial‐resolution multispectral or hyperspectral imagery from aircraft alongside the ALS data, and this can be used to map species (Dalponte, Bruzzone & Gianelle 2012) and some chemical components of tree leaves (Asner *et al*. 2015), allowing the WD term to be made species specific, just as it is in ground‐based inventories (Gonzalez *et al*. 2010). Recent technological advances mean that ALS acquisitions have a point density high enough to detect individual tree crowns (ITCs), and many crown delineation methods have been developed in the last years (Hyyppä *et al*. 2001; Ferraz *et al*. 2012; Eysn *et al*. 2015; Strîmbu & Strîmbu 2015), enabling such an approach (e.g. Yao, Krzystek & Heurich 2012; Breidenbach & Astrup 2014).

**Figure 1:**
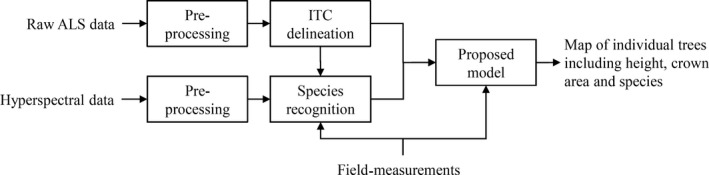
Architecture of the system in which the proposed method is included.

This paper sets out a methodological framework for tree‐centric biomass analysis (see Fig. 1) and illustrates the utility of the framework by analysing ALS and hyperspectral imagery from a 32‐km^2^ forest in the Italian Alps. We use a segmentation algorithm developed by us and allometric formulae provided by the Italian forest service (Scrinzi, Galvagni & Marzullo 2010; see Appendix S1 in Supporting information), but the framework is generic, and other segmentation algorithms and allometric formulae could be used if they outperform ours in a particular context. We show that tree‐centric airborne remote sensing (ARS) approaches deliver accurate high‐resolution maps of carbon density. While similar approaches have been advocated before (e.g. Omasa *et al*. 2003; Yao, Krzystek & Heurich 2012; Colgan, Asner & Swemmer 2013; Duncanson *et al*. 2014, 2015), we argue that rapid advances in technology now make them feasible over large spatial scales. We close the paper by discussing how the tree‐centric approach might be applied globally, including thoughts on how segmentation and species classification could be applied to more challenging types of forests, including multilayered tropical forests.

## Materials and methods

### Study Area Description and Field Data

The study area (32 km^2^) is located in the Italian Alps (Pellizzano, Trento), with an altitude range from 900 to 2200 m a.s.l. The forest is dominated by *Picea abies* (L.) Karst., with the presence of other coniferous species (e.g. *Abies alba* Mill., *Larix decidua* Mill., *Pinus cembra* L., *Pinus sylvestris* L. and *Pinus nigra* J.F.Arnold) and broadleaves species (e.g. *Populus tremula* L., *Betula* spp.). The forest is managed by selective logging, and trees harvested according to their stem diameter. At lower altitudes, the forest is more mixed and the structure is more complex, with the presence of multilayer forest, while at higher altitude the forest is sparse.

Field data used to calibrate and validate our tree‐centric ARS approach include three data sets (Dalponte & Coomes 2016):

1. *Angle‐count training plots* – Fifty‐two plots containing 2478 trees were used to calibrate the diameter estimation model and to train the classifier adopted for the tree species recognition. The 52 ACS plots were distributed using a stratified random sampling strategy. The species, DBH and position (bearing and distance from the plot centre) of all trees identified by a Haglöf angle prism (basal area factor equal to two) were measured (Table 1). Heights, measured for 156 of these trees using a Vertex hypsometer, were used to select site indices for each plot, and these were used to estimate height of all remaining trees using local allometric equations (Scrinzi, Galvagni & Marzullo 2010). Above‐ground biomass was obtained for all trees using local equations (Scrinzi, Galvagni & Marzullo 2010; Appendix S1).

**Table 1:** Statistics of the reference data from the 52 ACS plots used to build up the estimation models for the DBH and AGB

Species	*N*	AGB (kg)			DBH (cm)			Height (m)			Crown area (m^2^)		
		Min	Max	Mean	Min	Max	Mean	Min	Max	Mean	Min	Max	Mean
All	1762	3	7280	1079	6·5	121·0	49·4	3·5	48·8	28·1	1·5	55·4	30·9
*Abies alba*	70	43	2539	1095	15·5	77·0	47·9	12·4	39·6	27·8	12·0	53·9	34·6
Angiosperm	26	26	1330	485	13·5	54·5	32·3	7·3	31·5	22·5	8·6	46·6	28·2
*Larix decidua*	473	3	2971	1022	6·5	85·5	51·2	3·5	44·1	27·0	1·5	55·4	33·3
*Picea abies*	1174	7	7280	1124	8·0	121·0	49·3	4·4	48·8	28·9	1·7	54·9	29·9
*Pinus cembra*	19	13	997	447	10·5	75·5	38·5	7·8	16·1	12·9	6·0	37·6	18·1
2. *Individual tree training data set* – 3039 trees, distributed across the landscape, were used, in combination with the tree positions and species inside the 52 angle‐count sampling plots, to train and test the classifier used for the tree species recognition (Table 2). Tree species and positions were recorded for each tree.

**Table 2:** Statistics of the reference data used for the tree species classification

Species	Training		Test	
	Pixels	ITCs	Pixels	ITCs
*Abies alba*	1207	43	1340	42
Angiosperm	10 855	536	10 518	529
*Picea abies*	24 293	858	24 032	858
*Larix decidua*	13 248	379	12 213	379
*Pinus cembra*	743	57	687	56
*Pinus nigra*	470	17	482	16
*Pinus sylvestris*	171	3	59	3
3. *Validation plots* – 47 plots of 15 m radius randomly in the study area were used to validate the ITC delineation, and AGB and carbon density estimates. The DBH, species and height of all the trees within the plots (>4 cm DBH) were measured. The above‐ground biomass of each tree was estimated using the equations of (Scrinzi, Galvagni & Marzullo 2010; Appendix S1).

The positions of all plots and trees were precisely georeferenced using a differential GPS.

### Airborne Remote Sensing Data Collection and Pre‐processing

Airborne laser scanning data were acquired on 7–9 September 2012, using a Riegl LMS‐Q680i sensor (RIEGL Laser Measurement Systems GmbH, Horn, Austria). The scan frequency was 400 kHz and up to four returns were recorded. The average point density was of 48 pts m^−2^. A digital terrain model was extracted from the ALS points by the vendor and used to create a canopy height model (CHM) of the area. Hyperspectral data were acquired on 13 June 2013 with an AISA Eagle II sensor. Twenty‐one images were acquired in order to cover the whole study area. The minimum overlap among the images was 20%. Each image is characterized by 65 spectral bands acquired between 400 and 990 nm and by a spatial resolution of 1 m. The hyperspectral images were mosaicked in order to create a uniform image, and to reduce minor differences in reflectance occurring between the different images, the value of each pixel was normalized with respect to the sum of the original values of the same pixel in all the bands. From preliminary analyses, this operation resulted in a significant improvement of the final classification accuracies.

### ITCs Delineation

Individual tree crown delineation was conducted using an approach adapted from that of Hyyppä *et al*. (2001) which, despite its relative simplicity, came out among the best in a benchmark study comparing delineation methods across 18 sites in the Alps [method 2 in Eysn *et al*. 2015; Appendix S2; package *itcSegment* inside the software R (www.r-project.org)]. The ITC delineation approach finds local maxima within a rasterized CHM, designates these as tree tops and then uses a decision tree method to grow individual crowns around the local maxima. The approach goes through the following steps: (i) a low‐pass filter is applied to the rasterized CHM to smooth the surface and reduce the number of local maxima; (ii) local maxima are located using a circular moving window; a pixel of the CHM is labelled as local maxima if its value is greater than all other values in the window, provided that it is greater than some minimum height above‐ground; (iii) each local maximum is labelled as an ‘initial region’ around which a tree crown can grow; the heights of the four neighbouring pixels are extracted from the CHM and these pixels are added to the region if their vertical distance from the local maximum is less than some user‐defined percentage of the local maximum height, and less than some user‐defined maximum difference; this procedure is repeated for all the neighbours of cells now included in the region, and so on iteratively until no further pixels are added to the region; (iv) from each region that had been identified, the first‐return ALS points are extracted (having first removed low elevation points); and (v) a 2D convex hull is applied to these points, and the resulting polygons become the final ITCs. Note that this process is not completely automatic, as the size of the moving window, the small‐tree cut‐off height and the percentage and absolute height difference thresholds all need to be set by the user.

The delineated ITCs were automatically matched to the trees in all three field data sets. If only one field‐measured tree was included inside an ITC, then that tree was associated with that ITC. In the case of more than one field‐measured tree was included in a segmented ITC, the field‐measured tree with the height closer to the ITC height was chosen. We assessed the delineation accuracy by computing the detection rate (DET), omission error (OE = failure to detect a crown that exists), commission errors (CE = delineation of a crown that do not exist in reality) and accuracy index [AI = 100 − (OE + CE)] over the 47 fixed‐radius validation plots.

### Species Recognition

A support vector machines (SVM) classifier was used to identify species using features selected from the ALS and hyperspectral imagery. Tree species classification was carried out in two steps. Firstly, the sunlit pixels inside each ITC (Dalponte *et al*. 2014) were classified with the SVM, and secondly, the species of each ITC was decided by aggregating the classified pixels inside each ITC according to a majority rule. From the ALS data set, the 99th percentile of the first‐return points inside each ITC was used as a feature (if high‐point‐density ALS data are available, additional features can be extracted as showed in Dalponte, Bruzzone & Gianelle 2012), while 27 features were selected from the original hyperspectral data before classification using the sequential forward floating selection search algorithm (Pudil, Novovičová & Kittler 1994) and the Jeffries–Matusita distance metric (Bruzzone, Roli & Serpico 1995). We had already applied this approach successfully to similar forest types (Dalponte, Bruzzone & Gianelle 2012; Dalponte *et al*. 2014). The SVM implementation used was the one of the *kernlab* package in r software. The classification accuracy was assessed by computing the overall accuracy, kappa accuracy, mean class accuracy and the confusion matrix on a test set (see Table 2) and validation set (47 fixed‐radius plots).

### Individual Tree Biomass Estimated from ALS Data

AGB_TREE_ estimation of each ITC was done using the stem volume equations for temperate species of Scrinzi, Galvagni & Marzullo (2010) (Appendix S1) multiplied by the WD of the respective species (IPCC 2006). The AGB equation is similar to the generic formula of Chave *et al*. (2005, 2014) shown in eqn 1:

(eqn 4) $${\hat{\text{AGB}}}_{\mathrm{TREE}}=\alpha *{\text{WD}}^{\beta }*{\left(\text{DBH}-d_{0}\right)}^{\gamma }*H^{\delta }.$$

The values of α, β, γ, δ and *d*_0_ were taken from species‐specific tables (Scrinzi, Galvagni & Marzullo 2010). Note that the exponent of WD (β) is one, as also assumed by previous studies (Asner *et al*. 2012), while parameter δ ranges from 0·83 to 1·34 according to species (cf. Asner *et al*. 2012 assumed it to be 1). We do not have all information needed to estimate uncertainty in field biomasses, but DBH is typically measured with 1–2% accuracy and height with 5% accuracy in coniferous forests, in which case biomass uncertainty is about 6% (Chave *et al*. 2004). Using 456 trees in our 47‐plot validation data set, we added 6% random variation to field‐estimated AGB values and then used OLS regression to fit a line through field‐ vs. ALS‐estimated biomass values (log‐log‐transformed). We repeated this 100 times to gain estimates of the standard deviation of residuals as a proportion of AGB.

A nonlinear regression approach was used to model field‐based measurements of diameter (DBH in cm) with ALS‐derived measurements of crown area (CA in m^2^) and height (*H* in m) obtained from 1762 trees within the 52 angle‐count plots (these are the trees inside the 52 plots matching an ITC). The function we selected, after exploring many alternatives, was:

(eqn 5) $$\hat{\text{DBH}}=\varepsilon *H^{\rho }*\left(1+\vartheta *\text{CA}\right).$$

The height of each tree was defined as the 99th percentile of the first‐return ALS pulses inside the ITC polygon (used to reduce the effect of possible outliers), and crown area was calculated as the area of the ITC polygon. Species‐specific models were fitted for common species and a single model for all the less common ones. Models were parametrized using the *nlrq* function of quantile regression package *quantreg* in r (τ = 0·5), which is less sensitive to heteroscedasticity than conventional least‐square regression (Koenker & Park 1996).

### Plot‐level Estimates of Carbon Density

To test the effectiveness of the tree‐centric approach at estimating carbon density, we compared field‐estimated CD_PLOT_ with ARS‐estimated CD_PLOT_ within the 47 validation plots. Field‐based estimates were obtained by calculating the above‐ground biomasses of trees in a plot from their DBH, H and species (using eqn 4), summing to give total AGB, then multiplied by tree carbon content values (0·5 for conifers and 0·48 for angiosperms; IPCC, 2006; Thomas & Martin 2012) to give CD_PLOT_. ARS estimates were produced in a similar way, except that the biomasses of ITCs recognized from the ALS data were summed. Least‐squares regression was used to compare these estimates. Finally, the biomasses of all detected trees across the 32‐km^2^ area were estimated from their ITCs and used to produce two carbon density maps, one based on individual trees and one based on aggregating the ITC's carbon in squares of 100 × 100 size.

## Results

### ITC Delineation

**Figure 2:**
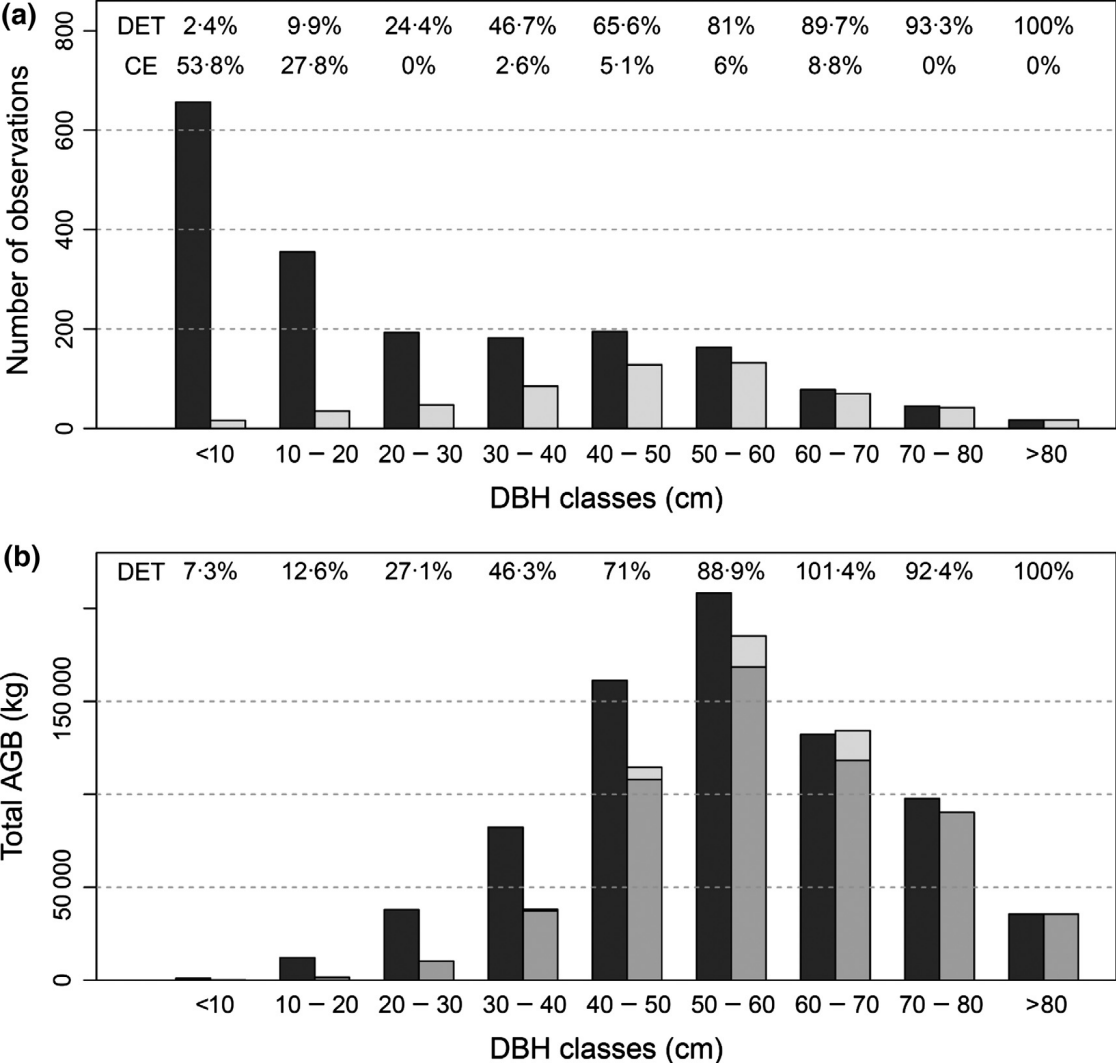
(a) total number of trees measured in plots and detected from airborne laser scanning, separated into diameter classes. The detection rate (DET) and the commission error (CE) in each diameter class are indicated; (b) total AGB (kg) measured in the field and detected in each diameter class. The dark grey bars refer to the field‐measured AGB, the grey ones to the AGB of the trees correctly matching between fields and airborne remote sensing (ARS) data, and the light grey ones to the AGB of all the ARS‐detected ones. At the top of the figure the percentage of biomass detected (DET) by the ARS approach respect to the field‐measured one.

Individual tree crown delineation was successful at detecting large trees but, as anticipated, failed to detect smaller trees in the understorey. The following analyses combine results from all 47 validation plots. In the largest stem diameter class (>80 cm DBH), all trees were correctly identified (100% DET) and no trees were incorrectly detected (i.e. 0% CE). However, DETs were much lower in the smaller size classes, while CEs became large (Fig. 2). Since small trees are much more numerous than larger trees, the overall DET was only 30·6% and the CE was 8·3%, with an AI of 22·3%. However, these small trees contribute little to biomass (Fig. 2), so detection failure has little effect on carbon density estimates (see later). Having only a small CE (especially for the large trees) is important, as compensating for such errors when estimating CD_PLOT_ is difficult.

There was a close relationship between field‐estimated heights and ALS‐estimated heights inside the 47 fixed‐radius plots: the RMSE was 2·3 m (*R* ^2^ of 0·90). ALS heights were in average 1% lower than field‐measured ones for big trees, perhaps because (i) laser pulses permeate into the canopy, (ii) the 99th percentile of ALS height was used as our measure of canopy height, and (iii) field‐estimated heights are measured with considerable uncertainty. The relationship between field‐measured and ALS‐estimated crown area was poor. A total of 198 trees within the 47 validation plots had field estimates of crown area and a matching delineated ITC. Comparison of field‐ vs. ALS‐estimated areas, by least‐squares regression, gave an RMSE of 17 m^2^ (the maximum detected crown size was 56 m^2^) and *R* ^2^ of 0·20 (see Appendix S4).

### Tree Species Classification

Within the test trees (trees in 52 ACS plots and another 3039 individuals; Table 2), the overall accuracy of the classification process was 82·4% with an average accuracy of 85·1%. Examining the confusion matrix (Table 3), it can be seen that *P. abies* (the dominant species) is mainly confused with *A. alba* and *L. decidua*, while the three pines are not confused with each other. Within the 47 validation plots, overall accuracy was 80·9%: the highest producer's accuracy (100%) was obtained for *A. alba,* while the dominant species (*P. abies*) got a producer's accuracy of 82·9%. The classification errors can arise for several reasons: imperfect matching of ITCs with ground data, trees having different spectral signatures at different stage of growth, isolated trees having ‘purer’ spectral signatures than trees within dense forests and species misidentification in the field.

**Table 3:** Confusion matrix, and accuracies at the individual tree crown level based on the test set

	*Abies alba*	Angiosperm	*Picea abies*	*Larix decidua*	*Pinus cembra*	*Pinus nigra*	*Pinus sylvestris*
*Abies alba*	32	2	46	7	0	0	0
Angiosperm	3	483	44	18	4	0	0
*Picea abies*	7	7	683	18	1	0	0
*Larix decidua*	0	36	83	334	10	2	0
*Pinus cembra*	0	1	2	0	41	0	0
*Pinus nigra*	0	0	0	2	0	14	0
*Pinus sylvestris*	0	0	0	0	0	0	3
Producer's accuracy (%)	76·2	91·3	79·6	88·1	73·2	87·5	100·0
Overall accuracy (%)	84·4						
Kappa accuracy	0·775						
Average accuracy (%)	85·1						

Grey highlighted cells show the number of correctly classified trees.

### DBH and AGB_TREE_ estimation

**Figure 3:**
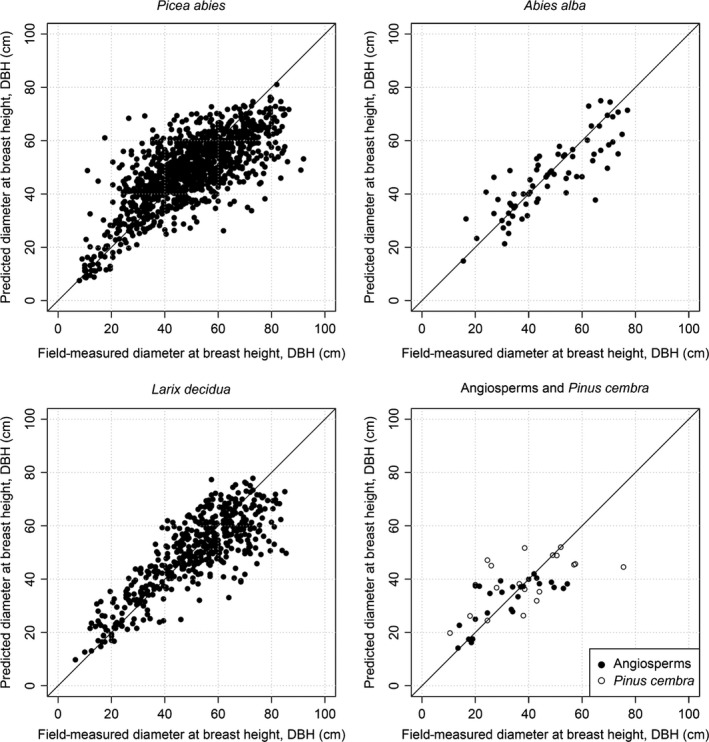
Estimation of the tree DBH for the field‐measured trees. Note that an outlier with DBH = 121 cm is omitted from the *Picea abies* panel.

**Figure 4:**
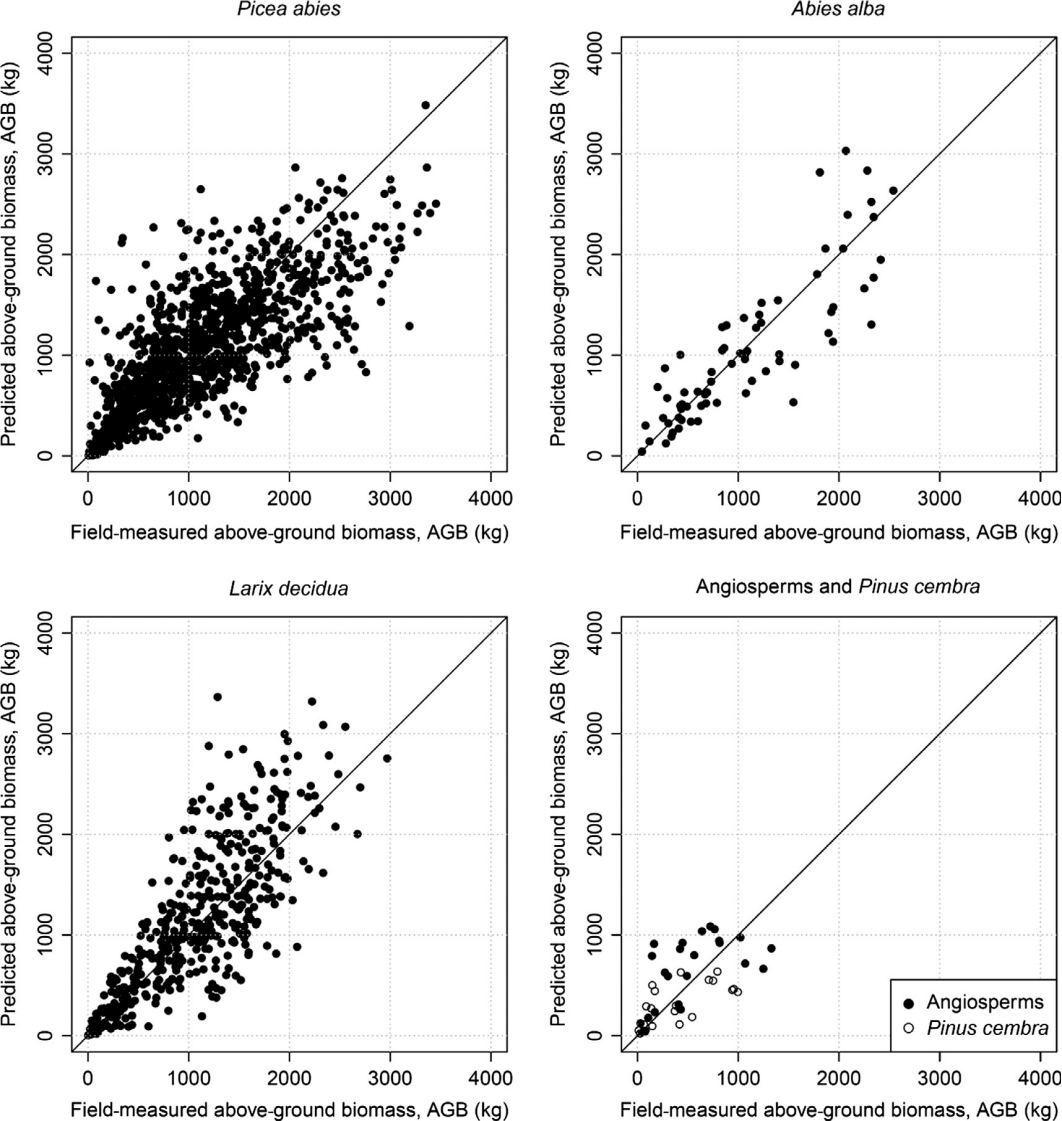
Estimation of the tree AGB on the field‐measured trees. Note that an outlier with AGB = 7200 kg is omitted from *Picea abies* panel.

**Figure 5:**
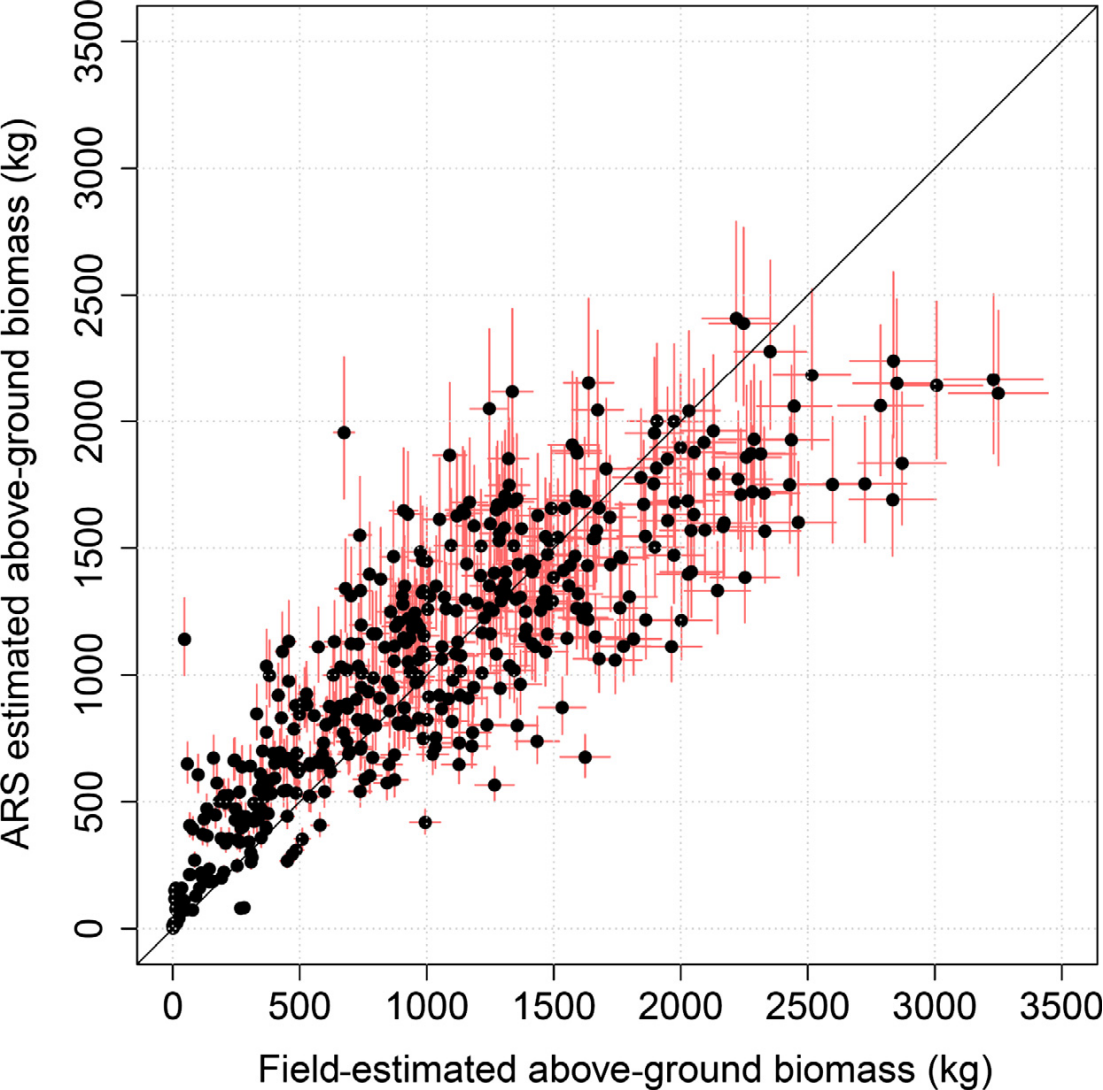
Field‐ vs. airborne remote sensing (ARS)‐estimated AGB of individual trees inside 47 validation plots. The error bars show standard errors, amounting to about 6% for the field estimates and 13% for ARS estimates.

Species‐specific coefficients of DBH estimation model (eqn 5) are shown in Table 4, and comparison of estimated vs. observed DBH of trees in the calibration data set is shown in Fig. 3. For trees represented by >100 samples, all coefficients have low standard errors and are significantly different from zero (Table 4); this demonstrates the value of including CA as well as *H* in the models. For these well‐replicated species, the DBH estimation equation had a better goodness‐of‐fit, and was less biased, when CA and *H* were included (Appendix S3). These species also had more accurate biomass estimation equations than the poorly replicated species (Fig. 4). The estimated biomasses of 456 trees in the validation plots are compared with field estimates in Fig. 5. A slight bias is evident, with the biomass of small trees overestimated and the biomass of large trees underestimated; the uncertainty of biomass estimates is about 13%.

**Table 4:** Coefficients (and standard errors) of DBH estimation model (eqn 5)

Species	ε		ρ		ϑ		RMSE (cm)
	Estimate	Std. Error	Estimate	Std. Error	Estimate	Std. Error	
All (1762)	**3·139**	**0·219**	**0·715**	**0·026**	**0·014**	**0·002**	11
*Abies alba* (70)	0·503	0·299	**1·287**	**0·219**	0·008	0·006	8·6
Angiosperms (26)	**3·745**	**1·640**	**0·631**	**0·181**	0·008	0·014	8·2
*Larix decidua* (473)	**4·695**	**0·447**	**0·553**	**0·041**	**0·021**	**0·004**	9·8
*Picea abies* (1174)	**2·102**	**0·289**	**0·848**	**0·047**	**0·011**	**0·002**	11·1
*Pinus cembra* (19)	1·362	3·668	1·303	1·119	0·001	0·017	12·9

The number of samples is given in parentheses and coefficients that are significantly different from zero are shown in bold. Root‐mean‐square errors are provided for each model.

### Carbon Density Estimation

**Figure 6:**
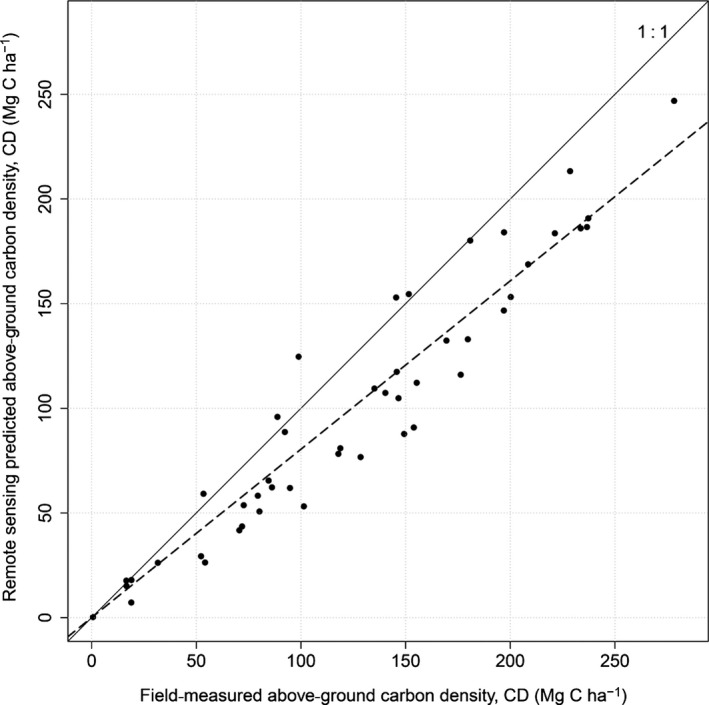
CD estimation over the 47 validation plots.

**Figure 7:**
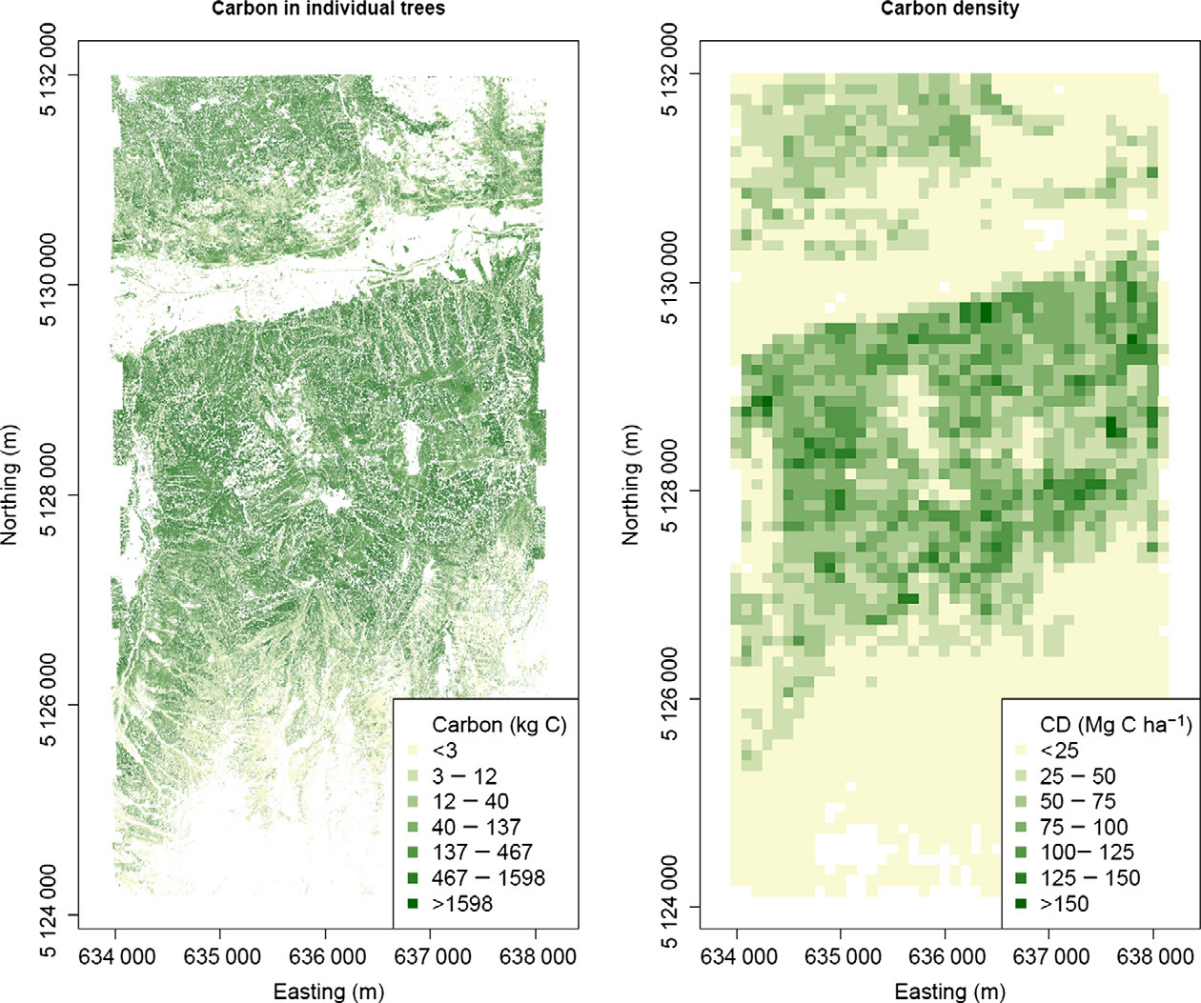
Carbon maps at individual tree crown level and within 100 × 100 m cells.

Aggregating the AGB_TREE_ estimates to the plot level increased the accuracy of the estimates. There was a close relationship between field‐ and ARS‐derived estimates of CD_PLOT_ (identical to the relationship between AGB_PLOT_ estimates). More than 98% of variation in field CD_PLOT_ is explained by ARS‐estimated CD_PLOT_ (adjusted *R* ^2^ = 0·98; Fig. 6). As expected, the field CD_PLOT_ is generally greater than the ARS‐estimated one, because small understorey trees have not been detected. This underestimation can be easily compensated with a hidden‐tree correction factor (here field CD_PLOT_ = 1·23 × ARS CD_PLOT_). The RMSE based on corrected values is 20 Mg C ha^−1^. Including crown area in the DBH estimation model led to a better goodness‐of‐fit than working with height alone. Repeating the analyses with just height, the adjusted *R* ^2^ is 0·96 and RMSE is 25 Mg C ha^−1^ (Appendix S3). Maps based on the carbon density of ITC or of cell can be generated (Fig. 7). These maps show the complete scalability of the proposed method, giving extremely high‐fidelity maps or aggregated number.

## Discussion

We have described a framework for estimating carbon density using a tree‐centric approach and illustrated the approach with data from the Italian Alps. The approach produced precise estimates of carbon stocks, with a systematic bias arising from undetected trees that we corrected using a multiplier (Fig. 6). However, given the complexity of ITC delineation approaches compared with classic estimation approaches, is the extra effort justified? We argue that the tree‐centric approach is worth pursuing for the following reasons: (i) it is similar in principle to ground‐based methods, so theoretically robust; (ii) individual wood densities can be included in calculations; and (iii) the information is completely scalable. These are discussed below.

Our approach is similar to the transparent and intuitive methods already established to obtain carbon densities from forest inventory plots, based on summing the masses of individual trees (e.g. Brown 1997; Coomes *et al*. 2001). Area‐based approaches lack this direct connection with field measurements because they are based on averaging information among trees within plots (Colgan, Asner & Swemmer 2013; Vincent, Sabatier & Rutishauser 2014). A study in South African savannas, which (uniquely) compared destructive sampling of trees with ALS and field surveys, found that a tree‐centric approach had similar accuracy to field inventory methods, and was twice as accurate as area‐based ALS analyses (Colgan, Asner & Swemmer 2013). Estimating tree volumes using terrestrial laser scanning (e.g. Calders *et al*. 2015) would provide an alternative way of comparing methods in regions where destructive sampling is impossible. Tree‐centric modelling improved the accuracy of biomass estimation in a mature conifer forests in California, but not in a broadleaf forest or pine a plantation in eastern USA, leading to the conclusion that allometric equations and delineation algorithms still need refinement (Duncanson *et al*. 2015). Expanding this approach to other sites will indeed require collection of new scaling relationships, so that wood volumes of individual trees can be estimated accurately from ALS. Synthesizing the allometries of 80 000 trees world‐wide, we find that a single metric – the product of a tree's height and crown diameter – is able to produce unbiased and accurate estimates of both stem diameter and above‐ground biomass (T. Jucker *et al*. unpublished data), so deriving a universal model is possible.

Recognition of species identities from hyperspectral data allowed individual tree biomasses to be calculated as the product of volume and WD, in contrast to most ALS approaches that use regionally averaged WD (Asner *et al*. 2012). This is potentially important because WD varies strongly along soil and climate gradients, and carbon maps derived from remote sensing data are strongly dependent upon the assumed form of that variation (Mitchard *et al*. 2014). A challenge with the ITC approach is that recognizing species by hyperspectral imaging remains difficult in diverse tropical forest. However, recent analyses from Amazon forest suggest that 1% of species hold 50% of carbon stocks (Fauset *et al*. 2015), so accurate carbon maps may only need a small fraction of abundant species to be identified. Given that hyperspectral leaf traits sometimes correlate with WD (Chave *et al*. 2006), it may be possible to infer WD from airborne hyperspectral imagery. Another possibility is to identify forests types from multispectral imagery (e.g. Dalponte, Bruzzone & Gianelle 2012), and use this information to refine carbon maps. However, hyperspectral data sets are better able to distinguish tree species (Dalponte, Bruzzone & Gianelle 2012) and can also be used to estimate a variety of physical and chemical leaf traits (Asner *et al*. 2015).

**Figure 8:**
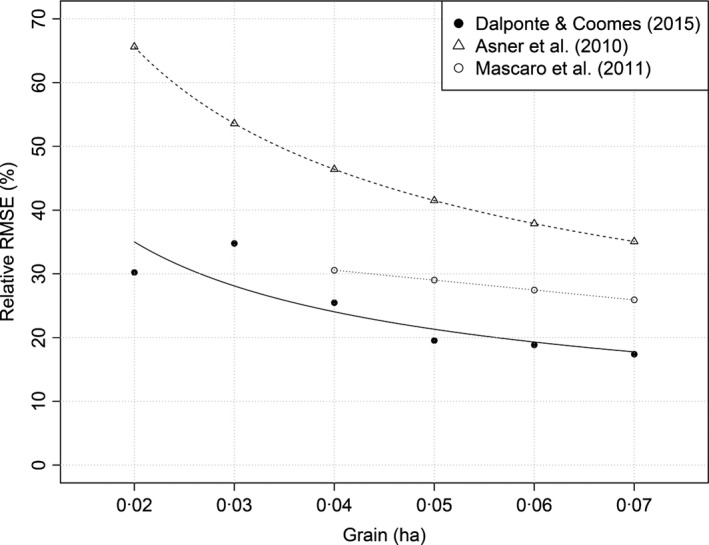
Observed decline in the prediction error of airborne laser scanning carbon density with decreasing spatial resolution using the tree‐centric method, compared with the theoretical expectation that errors should decline with (grain size)^−1/2^ (Asner *et al*. 2010), and with the results obtained by Mascaro *et al*. (2011). The relative RMSE has been computed as the ratio between RMSE and the mean CD of the plots, multiplied by 100. The RMSE of Mascaro *et al*. (2011) was extracted from Fig. 3 of that paper; the RMSE of Asner *et al*. (2010) has been computed from the equation contained in the Supporting Information of that paper.

The tree‐centric approach is less sensitive to edge effects than classic approaches. When using area‐based approaches, edge effects arise when a large tree which is just outside a plot's boundary is not included in the field‐based biomass calculation, but much of its crown lies within the plot and so it influences the canopy top height and ALS estimate of biomass (Mascaro *et al*. 2011). They also arise when trees included in the ground plots do not appear in the ALS plot (or vice versa), perhaps because the corners of plots have been geolocated inaccurately, or because edge trees are leaning so that trunks and crown centres are not aligned. Uncertainty arising from edge effects is reduced by establishing larger ground plots (Mascaro *et al*. 2011). A plot of 0·07 ha (i.e. the size of our validation plots) has an RMSE of only 18% (Fig. 8), compared with 35% reported by Asner *et al*. (2012) for tropical forests, or 25% when methods are applied to reduce edge effects. These comparisons need to be treated with caution, as alpine forests are very different in structure to tropical forest. Nevertheless, the tree‐centric approach is relatively insensitive to plot size – we estimate RMSE = 30% for 0·02‐ha plots compared with 65% in Asner *et al*. (2012) – because the only source of edge error is inaccuracy in deciding whether tree centres are inside or outside of boundaries.

Finally, the new proposed approach is flexible because – as shown in Fig. 7 – carbon can be mapped at any scale from single trees to whole regions. Since estimation does not depend on a specific plot size, there are fewer constraints on field data collection: calibration trees can be collected in any kind of plot, with any kind of strategy, so long as samples are representative in terms of species and size ranges. This makes it possible to use field data collected for other purposes when calibrating.

### Towards a Universal Tree‐centric Mapping Approach

While tree‐centric approaches hold great promise, particularly given the rapid advancement of technology, some key issues remain to be overcome. A key advantage of the approach is that species information allows specific allometries to be used in calculations, but very real difficulties remain in reliable species identification from hyperspectral imagery. A second issue is that inclusion of crown area into biomass estimation equations leads to improvements in accuracy, but ALS and field estimates of crown area were only weakly correlated. It seems likely that inaccurate field estimates are responsible, as measuring crown widths in N–S and E–W directions is a basic approach, and because tests with a different approach to tree delineation, which works with the entire point cloud, yield similar results to ours (Lee 2016). A final issue is that ITC recognition approaches based on CHMs fails to detect small trees hidden beneath the upper canopy. Although we corrected for this bias using a multiplier, it is very likely that the multiplier will vary among forest types that differ in complexity, meaning that local calibration is required to map carbon accurately. This calibration can be carried out using a semi‐ITC approach where the percentage of missing trees is estimated from ALS data (Breidenbach & Astrup 2014). The development of methods that use the entire ALS point cloud or waveform data, instead of just the CHM, to improve the detection of understorey trees may provide a solution to this problem (Strîmbu & Strîmbu 2015). ALS, hyperspectral sensing and computational power are all advancing rapidly, making it increasingly feasible to use ITC approaches for effective mapping of forest carbon density.

## Data accessibility

Data deposited in the Dryad repository: http://datadryad.org/resource/doi:10.5061/dryad.hf5rh (Dalponte & Coomes, 2016).

## Supporting information


**Appendix S1.** Allometric equations for AGB estimation.
**Table S1‐1.** Coefficients of the allometric equations of Scrinzi, Galvagni & Marzullo (2010) and wood densities (WD) from (IPCC 2006). The wood density (WD) is expressed in kg m^−3^.
**Appendix S2.** Individual tree crowns delineation method.
**Appendix S3.** Tree DBH and AGB estimation and carbon density estimation at tree and plot level using only the height information.
**Table S3‐1.** Coefficients of the models used for the estimation of the DBH (equation S3‐1)
**Figure S3‐1.** Estimation of the tree DBH for the field measured trees. NB: in the ‘*Picea abies*’ graph there is an outlier with 121 cm diameter not showed in the graph.
**Figure S3‐2.** Estimation of the tree AGB on the field measured trees. NB: in the ‘*Picea abies*’ graph there is an outlier with 7200 kg of AGB not showed in the graph.
**Figure S3‐3.** CD estimation over the 47 validation plots.
**Appendix S4.** Field‐ and ALS‐estimated crown areas.
**Figure S4‐1.** Field‐ vs. ALS‐estimated crown areas. The dashed line is representing the Type II regression line (RMA) among them.
