# Supplementary material for: Tree‐centric mapping of forest carbon density from airborne laser scanning and hyperspectral data
Source: Methods Ecol Evol. 2016 May 14;7(10):1236–45. doi: 10.1111/2041-210X.12575 (PMC5137341; doi:10.1111/2041-210X.12575)
Supplement: Supplementary file 1 — Appendix S1. Allometric equations for AGB estimation. Table S1‐1. Coefficients of the allometric equations of Scrinzi, Galvagni & Marzullo (2010) and wood densities (WD) from (IPCC 2006). The wood density (WD) is expressed in kg m−3. Appendix S2. Individual tree crowns delineation method. Appendix S3. Tree DBH and AGB estimation and carbon density estimation at tree and plot level using only the height information. Table S3‐1. Coefficients of the models used for the estimation of the DBH (equation S3‐1) Figure S3‐1. Estimation of the tree DBH for the field measured trees. NB: in the ‘Picea abies’ graph there is an outlier with 121 cm diameter not showed in the graph. Figure S3‐2. Estimation of the tree AGB on the field measured trees. NB: in the ‘Picea abies’ graph there is an outlier with 7200 kg of AGB not showed in the graph. Figure S3‐3. CD estimation over the 47 validation plots. Appendix S4. Field‐ and ALS‐estimated crown areas. Figure S4‐1. Field‐ vs. ALS‐estimated crown areas. The dashed line is representing the Type II regression line (RMA) among them. [file MEE3-7-1236-s001.docx]

# Supporting Information

##

The allometric equations used for the $\hat{AGB}_{TREE}$ estimation are based on the equations published in Scrinzi *et al*. (2010) multiplied by the wood density ($WD$) of each species (IPCC 2003). The equation $\bar{AGB}_{TREE}$ is in the following form:

| $\hat{AGB}_{TREE} =WD*\alpha*{(DBH-d_{0})}^{\gamma}*H^{\delta}$ | (S1-1) |
| --- | --- |

where $DBH$ is the diameter in centimetres, $H$ the height in meters, and $\hat{AGB}_{TREE}$ is the estimated above ground biomass in kilograms. The coefficients used for the different species are in Table S1-1.

Table S1-1. Coefficients of the allometric equations of Scrinzi *et al*. (2010) and wood densities (WD) from (IPCC 2003). The wood density (WD) is expressed in kg/m^3^.

|  | WD | $\alpha$ | $\gamma$ | $\delta$ | *d_0_* |
| --- | --- | --- | --- | --- | --- |
| Abies alba | 400 | 0.000163 | 1.70656 | 0.941905 | 3.69465 |
| Angiosperms | 580 | 0.000055 | 1.942089 | 1.00642 | 4.0091 |
| Larix decidua | 460 | 0.000108 | 1.407756 | 1.341377 | 3.69465 |
| Picea abies | 400 | 0.000177 | 1.564254 | 1.051565 | 3.69465 |
| Pinus cembra | 420 | 0.000188 | 1.613713 | 0.985266 | 3.69465 |
| Pinus nigra | 420 | 0.000129 | 1.763086 | 0.938445 | 3.69465 |
| Pinus sylvestris | 420 | 0.000102 | 1.918184 | 0.830164 | 3.69465 |

## Appendix S2. INDIVIDUAL TREE CROWNS DELINEATION METHOD

The method used for the ITC delineation is based on that of Hyyppä *et al*. (2001). Hyyppä et al. exploits a raster canopy height model (CHM) on which local maxima are detected, and then they growth regions starting from these local maxima. Differently, the method used in this paper uses both a CHM in raster format, and the ALS point cloud with normalized z. In greater detail the steps of the method are the following:

- - - 1. a low-pass filter (LPF) is applied to the raster image of the CHM;
      2. local maxima $S=\left\{ s_{1},\ldots,s_{N} \right\}$ are defined using a moving window. A CHM pixel $CHM(x,y)$ is a local maxima point if:

| $CHM\left( x,y \right)\in S if \left\{ \begin{matrix} CHM\left( x,y \right)=\max(moving window) \\ CHM\left( x,y \right)>S_{TH} \end{matrix} \right.$ | (S2-1) |
| --- | --- |

where $S_{TH}$ is a minimum height threshold fixed by the user;

- - - 1. initial regions are defined starting from the local maxima points. A label map $L$ is defined:

| $\left\{ \begin{matrix} L_{i,j}=k if CHM(i,j)\in S \\ L_{i,j}=0 if CHM(i,j)\notin S \end{matrix} \right.$ | (S2-2) |
| --- | --- |

where $k$ is a unique identifier for each local maxima.

- - - 1. starting from $L$, regions growth according to the following procedure:
  1. a label map point $L_{i,j}\neq0$ is considered and its four neighbor pixels ($NP$) in the CHM are extracted:

| $NP=\left\{ CHM\left( i,j-1 \right);CHM\left( i-1,j \right);CHM\left( i,j+1 \right);CHM\left( i+1,j \right) \right\}$ | (S2-3) |
| --- | --- |

- 1. a neighbor pixel $NP\left( i^{'},j^{'} \right)$ is added to the region $n$ if:

| $\left\{ \begin{matrix} dist\left( NP\left( i^{'},j^{'} \right),L_{i,j} \right)<DistMax \\ NP\left( i^{'},j^{'} \right)>\left( {CHM}_{i,j}*PercThresh \right) \\ L_{i^{'},j^{'}}\neq0 \end{matrix} \right.$ | (S2-4) |
| --- | --- |

where $PercThresh\in\left( 0;1 \right)$, and $DistMax>0$. These parameters are fixed by the user;

- 1. this procedure is iterated over all the pixels that have $L_{i,j}\neq0$, and it is repeated until no pixels are added to any region;
     - 1. from each region in $L$ the first return ALS points are extracted. At this stage it is important to remove from each region low elevation points and thus an automatic Otsu thresholding (Otsu, 1979) is applied to the normalized heights of the ALS points, like in (Ene *et al*., 2012);
       2. the first return ALS points higher than the Otsu threshold are taken and a 2D convex hull is applied to these points;
       3. the resulting polygons are the final ITCs.

The method described above was coded in R by the authors and it can be found in the R package *itcSegment*. In this study we used a CHM with 0.5 m spatial resolution, a 3x3 LPF filter, a 5x5 searching window for local maxima, $S_{TH}$ equal to 2 m,$DistMax$ equal to 30 pixels, and $PercThresh$ equal to 0.7.

## Appendix S3. TREE DBH AND AGB ESTIMATION AND CARBON DENSITY ESTIMATION AT TREE AND PLOT LEVEL USING ONLY THE HEIGHT INFORMATION

In case the crown area (CA) is poorly estimated or impossible to retrieve, the DBH can be estimated with a simplified version of the proposed equation (Equation 5) based only on the height information:

| $\hat{DBH}=\varepsilon*H^{\rho}$ | (S3-1) |
| --- | --- |

where $\hat{DBH}$ is the estimated diameter in centimetres, $H$ is the height in meters. Species specific models were parametrized using the *nlrq* function of quantile regression package *quantreg* in R (tau = 0.5) and the coefficients along with their standard errors are showed in Table S3-1.

Table S3-1. Coefficients of the models used for the estimation of the DBH (equation S3-1).

| Species | $\varepsilon$ | | $\rho$ | |
| --- | --- | --- | --- | --- |
|  | Estimate | Std. Error | Estimate | Std. Error |
| All | 2.175 | 0.119 | 0.930 | 0.017 |
| *Abies alba* | 0.373 | 0.180 | 1.456 | 0.144 |
| Angiosperms | 3.404 | 1.383 | 0.728 | 0.132 |
| *Larix decidua* | 3.418 | 0.366 | 0.812 | 0.031 |
| *Picea abies* | 1.526 | 0.223 | 1.027 | 0.043 |
| *Pinus cembra* | 1.285 | 2.031 | 1.332 | 0.600 |

Using equation S3-1 and the coefficients of Table S3-1 the DBH of each detected tree was estimated using as species information the species estimated with the hyperspectral data. Afterwards the AGB has been estimated using allometric equations (Scrinzi *et al*., 2010) multiplied by the wood density ($WD$) of each species (IPCC 2003) (see Appendix S1). In Figure SI3-1 and SI3-2 DBH and AGB estimation results at ITC level are showed.

The field-estimated CD_PLOT_ was compared with ARS-estimated CD_PLOT_ within the 47 validation plots. Field-based estimates were obtained by calculating the above-ground biomasses of trees in a plot from their DBH, H and species (using equation 4 of the paper), summing to give total AGB, then multiplied by tree carbon content values (0.5 for conifers and 0.48 for angiosperms; IPCC, 2006; Thomas & Martin, 2012) to give CD_PLOT_. Remote sensing estimates were produced in a similar way, except that the biomasses of ITCs recognised from the ALS data were summed. Least-squares regression was used to compare these estimates. There was a close relationship between field- and ARS-derived estimates of CD_PLOT_. More than 96% of variation in field CD_PLOT_ is explained by ARS estimated CD_PLOT_ (Adjusted-R^2^ = 0.96; Figure S3-3). The RMSE based on bias corrected values was 25 Mg C ha^-1^.


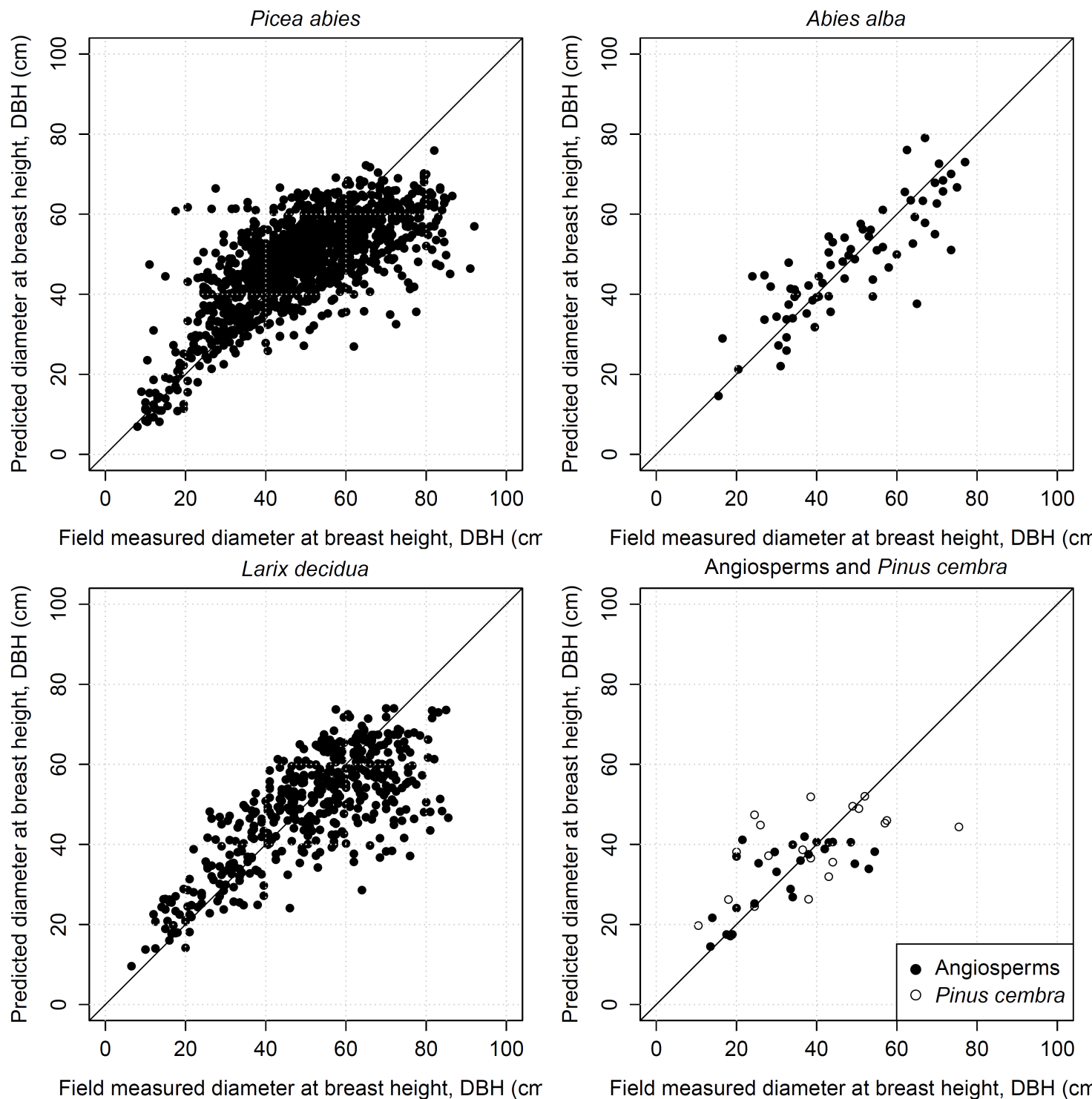


Figure S3-1. Estimation of the tree DBH for the field measured trees. NB: in the “*Picea abies*” graph there is an outlier with 121 cm diameter not showed in the graph.


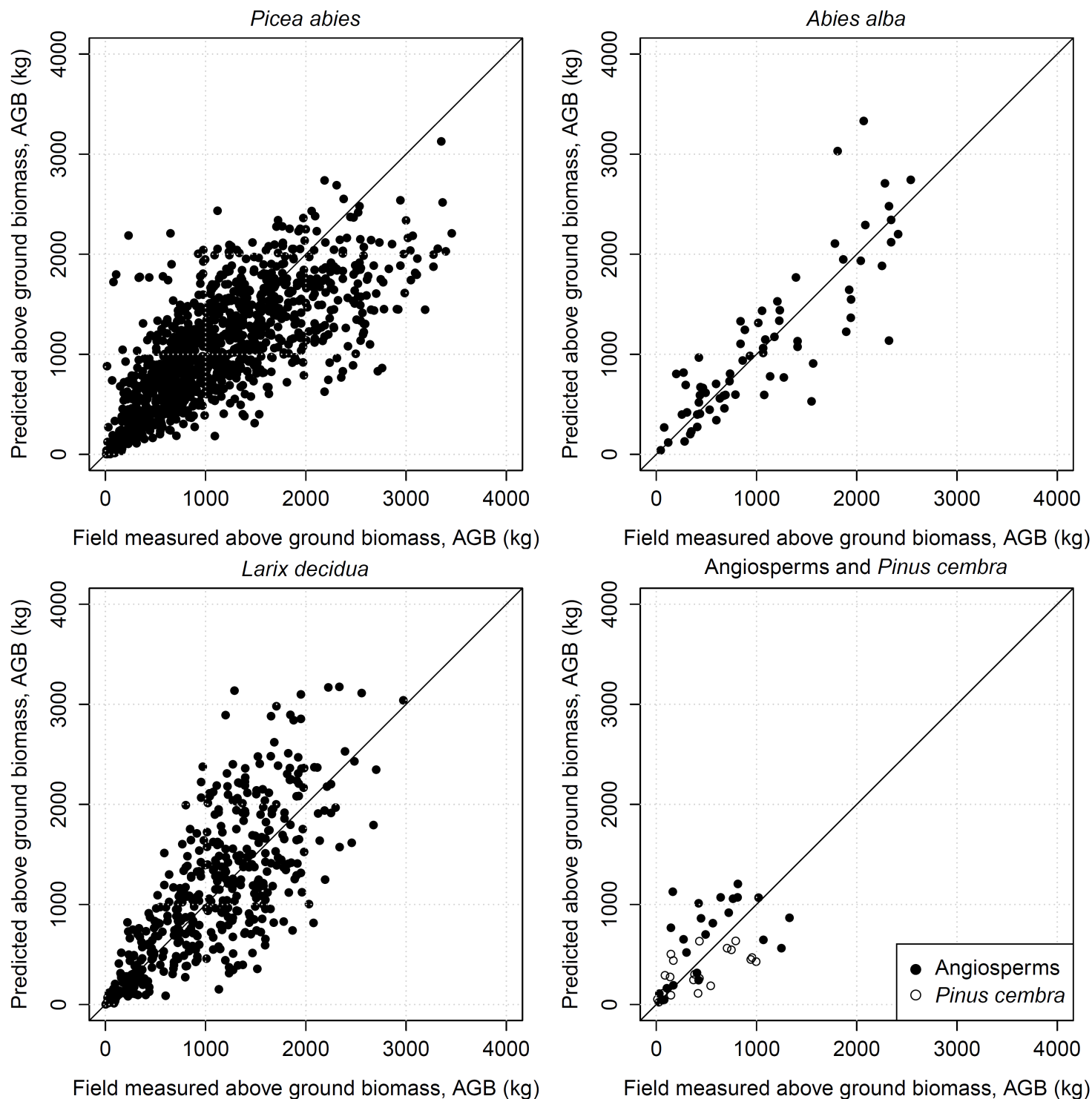


Figure S3-2. Estimation of the tree AGB on the field measured trees. NB: in the “*Picea abies*” graph there is an outlier with 7200 kg of AGB not showed in the graph.


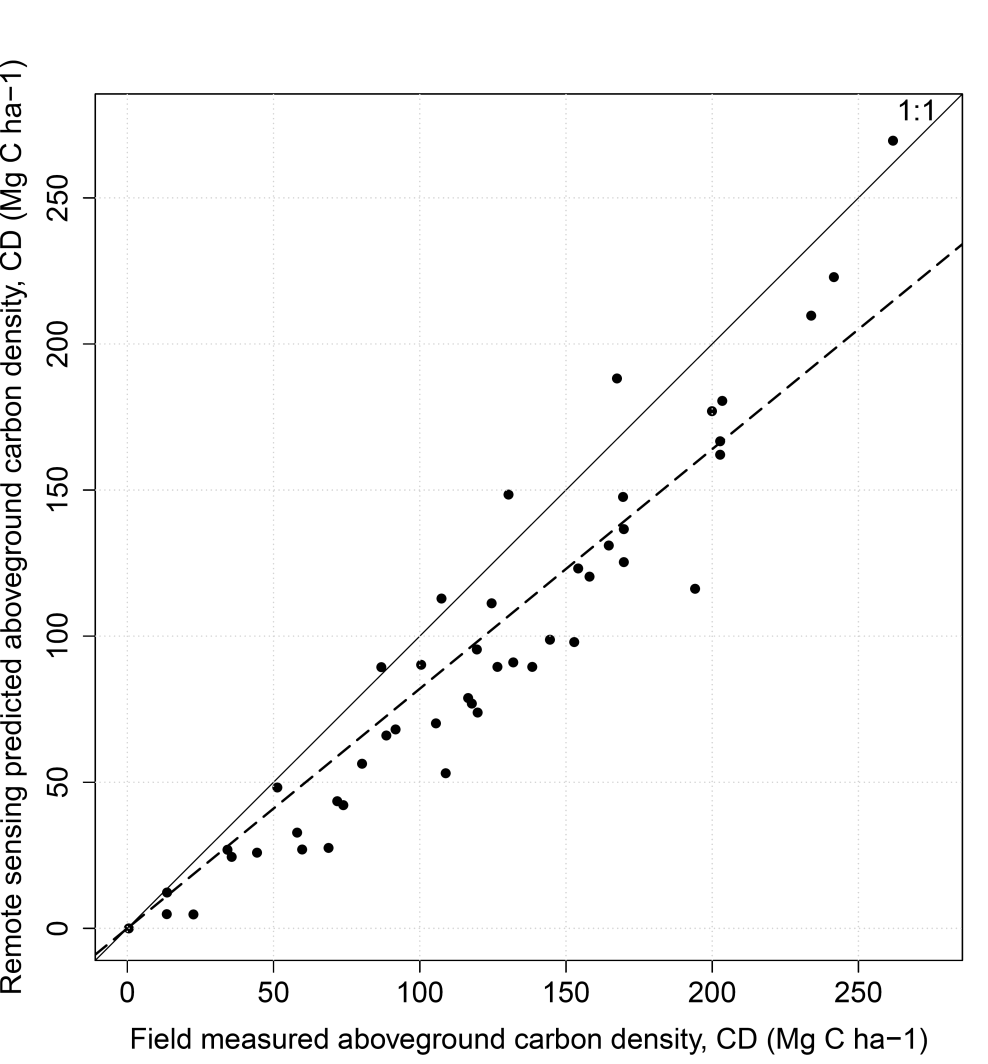


Figure S3-3. CD estimation over the 47 validation plots.

## Appendix S4. FIELD- AND ALS-ESTIMATED CROWN AREAS

In Figure S4-1 the relationship between field- and ALS-estimated crown areas is showed. The dashed line is representing the relationship obtained with a Type II regression (RMA).


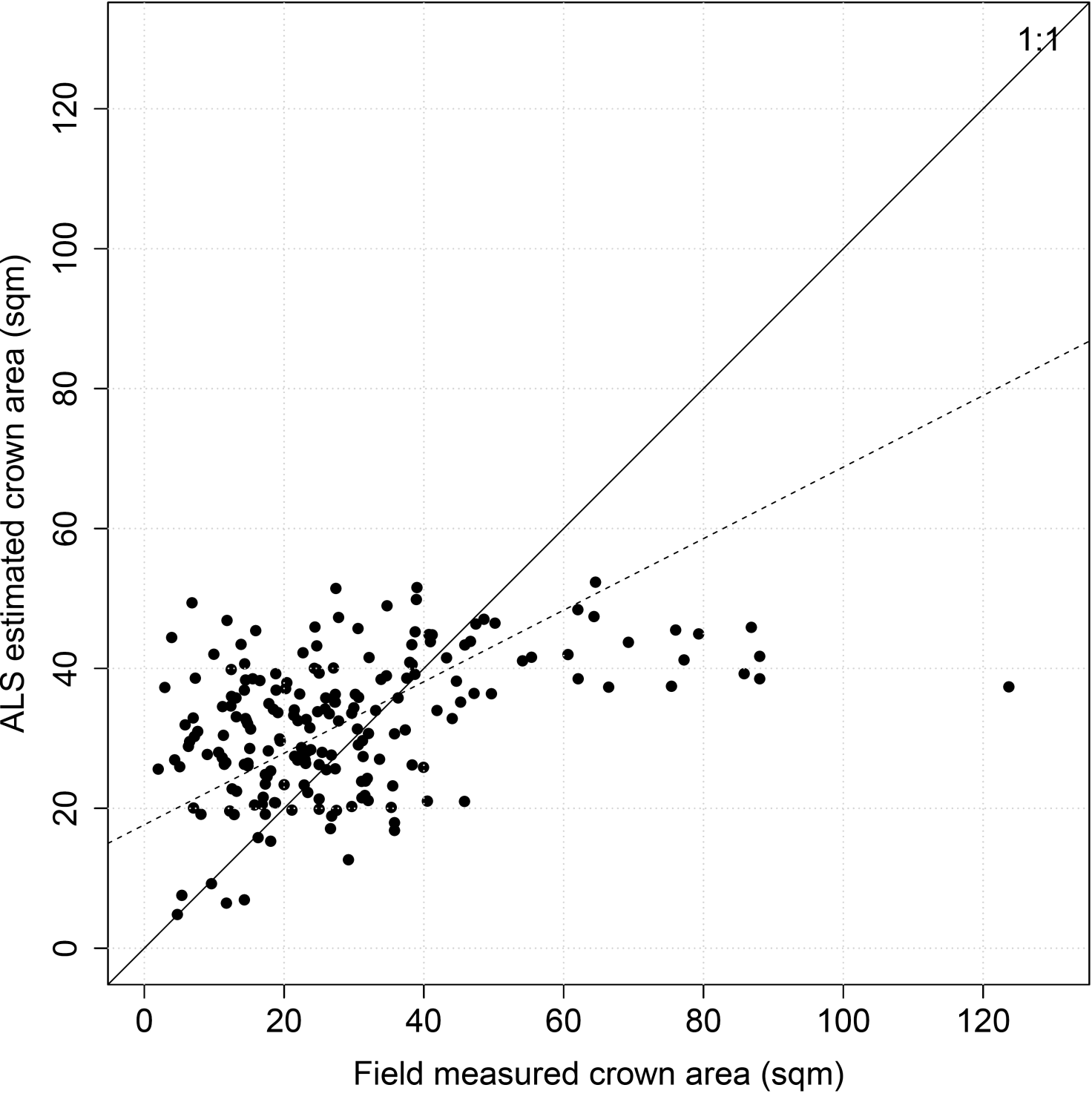


Figure S4-1. Field- versus ALS-estimated crown areas. The dashed line is representing the Type II regression line (RMA) among them.
